# Deviations of the immune cell landscape between healthy liver and hepatocellular carcinoma

**DOI:** 10.1038/s41598-018-24437-5

**Published:** 2018-04-18

**Authors:** Nataliya Rohr-Udilova, Florian Klinglmüller, Rolf Schulte-Hermann, Judith Stift, Merima Herac, Martina Salzmann, Francesca Finotello, Gerald Timelthaler, Georg Oberhuber, Matthias Pinter, Thomas Reiberger, Erika Jensen-Jarolim, Robert Eferl, Michael Trauner

**Affiliations:** 1 Division of Gastroenterology and Hepatology, Department of Internal Medicine III, Medical University of Vienna, Waehringer Guertel 18-20, A-1090 Vienna, Austria; 2 Centre for Medical Statistics, Informatics and Intelligent Systems, Medical University of Vienna, Spitalgasse 23, A-1090 Vienna, Austria; 3 Institute of Cancer Research, Internal Medicine I, Medical University of Vienna and Comprehensive Cancer Center (CCC), Borschkegasse 8a, A-1090 Vienna, Austria; 4 Clinical Institute of Pathology, Medical University of Vienna, Waehringer Guertel 18-20, A-1090 Vienna, Austria; 5 Institute of Pathophysiology and Allergy Research, Center of Pathophysiology, Infectiology and Immunology, Medical University of Vienna, Vienna, Austria; 6 Division of Bioinformatics, Biocenter, Medical University of Innsbruck, Innrain 80-82, 6020 Innsbruck, Austria; 7 Comparative Medicine, The Interuniversity Messerli Research Institute of the University of Veterinary Medicine Vienna, Medical University Vienna and University Vienna, Vienna, Austria

## Abstract

Tumor-infiltrating immune cells are highly relevant for prognosis and identification of immunotherapy targets in hepatocellular carcinoma (HCC). The recently developed CIBERSORT method allows immune cell profiling by deconvolution of gene expression microarray data. By applying CIBERSORT, we assessed the relative proportions of immune cells in 41 healthy human livers, 305 HCC samples and 82 HCC adjacent tissues. The obtained immune cell profiles provided enumeration and activation status of 22 immune cell subtypes. Mast cells were evaluated by immunohistochemistry in ten HCC patients. Activated mast cells, monocytes and plasma cells were decreased in HCC, while resting mast cells, total and naïve B cells, CD4^+^ memory resting and CD8^+^ T cells were increased when compared to healthy livers. Previously described S1, S2 and S3 molecular HCC subclasses demonstrated increased M1-polarized macrophages in the S3 subclass with good prognosis. Strong total immune cell infiltration into HCC correlated with total B cells, memory B cells, T follicular helper cells and M1 macrophages, whereas weak infiltration was linked to resting NK cells, neutrophils and resting mast cells. Immunohistochemical analysis of patient samples confirmed the reduced frequency of mast cells in human HCC tumor tissue as compared to tumor adjacent tissue. Our data demonstrate that deconvolution of gene expression data by CIBERSORT provides valuable information about immune cell composition of HCC patients.

## Introduction

Hepatocellular carcinoma (HCC) represents a leading cause of cancer mortality worldwide^1^. Therapeutic options include tumor resection or ablation, transarterial chemoembolisation, liver transplantation and treatment with the tyrosine kinase inhibitor sorafenib^2^. However, HCC is often diagnosed at advanced disease stages that allow only palliative treatments. Therefore, investigation of new therapeutic approaches in HCC is required.

Immunotherapy with immune checkpoint inhibitors is clinically approved for treatment of melanoma, non-small cell lung cancer, renal and bladder cancers^3^. Extension of this therapeutic concept to other malignancies including HCC is currently focus of basic and clinical research^4–7^. The immune phenotype is a relevant prognostic factor in various tumors^8,9^. The degree and distribution of immune cell infiltration might also stratify patients into responders and non-responders to anticancer therapies^8,10–12^.

Immunohistochemistry (IHC) and flow cytometry are common techniques to analyze the immune cell composition of tumors but these techniques have limitations. Only few immune cell types can be evaluated at once by IHC and the unambiguous assignment of certain cell types by flow cytometry is usually based on several marker proteins, which is limited by the number of fluorescence channels. The systems biology tool CIBERSORT employs deconvolution of bulk gene expression data and a sophisticated algorithm for *in silico* quantification of many immune cell types in heterogeneous samples as tumor stroma^13^. Gene expression data can be obtained for a huge number of tumor samples, which allows identification of immune cell-based prognostic and therapeutic markers by CIBERSORT after stratification into molecular subtypes.

High resolving power is a key benefit of CIBERSORT, which enumerates 22 immune cell types at once and applies signatures from ~500 marker genes to quantify the relative fraction of each cell type^13^. The method was successfully validated by FACS and used for determination of the immune cell landscapes in several malignant tumors such as colon, lung and breast^9,13–15^.

Here, we used CIBERSORT for deconvolution of global gene expression data to define the immune cell landscape of healthy human livers, HCC and HCC-adjacent tissues. Our data also uncovered distinct immune phenotypes for molecular HCC subclasses.

## Results

### Adaptive immune cells in HCC

**Figure 1:**
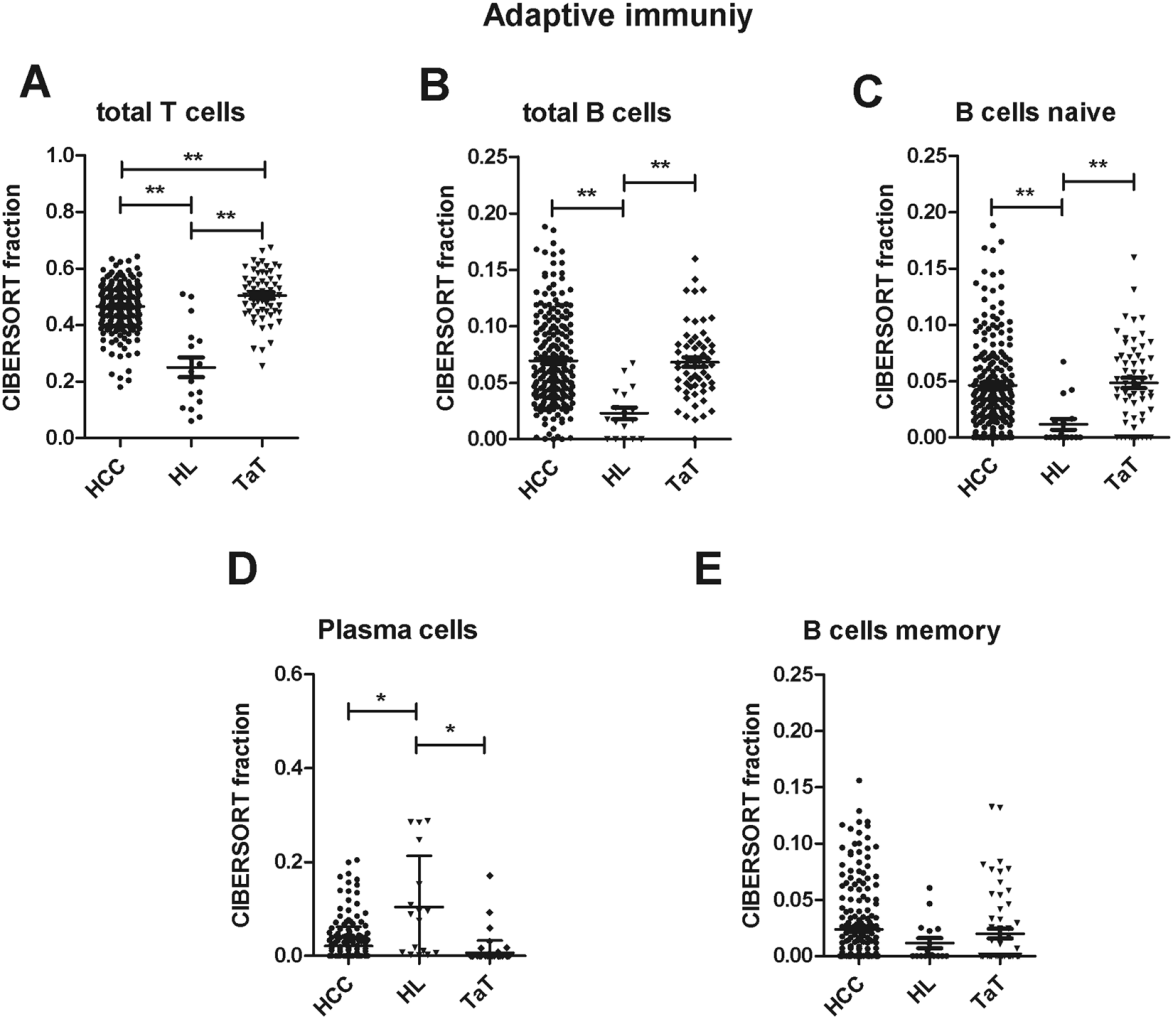
Adaptive immunity cells in human HCC tumor tissue (HCC), adjacent tissue (TaT) and healthy. liver (HL). CIBERSORT immune cell fractions were determined for each patient; each dot represents one patient. Mean values and standard deviations for each cell subset including total T cells (**A**), total B cells (**B**), naïve B cells (**C**), plasma cells (**D**) and memory B cells (**E**) were calculated for each patient group and compared using one-way ANOVA. *p < 0.05; **p < 0.01.

The fraction of total T cells, B cells and naïve B cells was higher in HCC and HCC adjacent tissue (TaT) than in healthy liver tissue (Fig. 1A–C, Table 1). TaT contained even more T cells than HCC (Fig. 1A). Plasma cells were mainly present in healthy livers and less frequent in HCC and TaT (Fig. 1D). Memory B cells were not significantly altered between tissues (Fig. 1E).

**Table 1:** Comparison of CIBERSORT immune cell fractions between HCC, HL and TaT.

Immune cell type	CIBERSORT fraction in % of all infiltrating immune cells					
	mean ± SD			p-values (with Bonferroni correction)		
	HCC	HL	TaT	HCC vs HL	HCC vs TaT	TaT vs HL
T cells total	0.466 ± 0.081	0.250 ± 0.146	0.505 ± 0.088	4e-19	8e-3	1e-21
T cells CD8^+^	0.125 ± 0.067	0.060 ± 0.102	0.157 ± 0.065	2e-3	9e-3	1e-5
T cells CD4^+^ memory resting	0.224 ± 0.088	0.079 ± 0.057	0.248 ± 0.090	2e-8	0.205	1e-9
T cells CD4^+^ memory activated	0.031 ± 0.033	0.003 ± 0.007	0.024 ± 0.033	6e-3	0.507	8e-2
T cells Follicular Helper	0.077 ± 0.052	0.024 ± 0.037	0.048 ± 0.043	6e-4	5e-4	0.327
Tregs	0.010 ± 0.019	0.024 ± 0.035	0.026 ± 0.034	0.136	9e-5	1
T cells gamma delta	0.007 + 0.018	0.025 + 0.050	0.002 + 0.007	2e-3	0.346	2e-4
B cells total	0.070 ± 0.041	0.023 ± 0.022	0.068 ± 0.032	6e-6	1	7e-5
B cells memory	0.025 ± 0.035	0.010 ± 0.02	0.020 ± 0.033	0.328	0.865	1
B cells naïve	0.048 ± 0.040	0.013 ± 0.021	0.048 ± 0,037	4e-3	1	6e-3
Macrophages total	0.271 ± 0.070	0.173 ± 0.097	0.241 ± 0.065	3e-7	0.013	7e-2
M0 macrophages	0.010 ± 0.023	0.029 ± 0.052	0.011 ± 0.018	0018	1	6e-2
M1 macrophages	0.091 ± 0.036	0.032 ± 0.030	0.100 ± 0.039	7e-8	3e-1	4e-9
M2 macrophages	0.173 ±± 0.074	0.093 ± 0.086	0.129 ± 0.060	2e-4	2e-4	0,265
Mast cells resting	0.050 ± 0.052	0.006 ± 0.020	0.071 ± 0.061	1e-2	6e-2	2e-4
Mast cells activated	0.010 ± 0.022	0.204 ± 0.199	0.005 ± 0.011	5e-31	1	2e-29
Neutrophils	0.041 ± 0.034	0.078 ± 0.070	0.034 ± 0.022	0,103	1	0,674
Dendritic cells resting	0.012 ± 0.021	0.003 ± 0.005	0.017 ± 0.023	0.354	0.363	0.073
Dendritic cells activated	0.002 ± 0.005	0.003 ± 0.006	0.0 ± 0.0	1	0.080	0.204
Monocytes	0.009 ± 0.0130	0.084 ± 0.083	0.007 ± 0.011	5e-24	1	9e-23
Eosinophils	0.007 ± 0.016	0.012 ± 0.028	0.003 ± 0.007	1	0.1336	0.103

**Figure 2:**
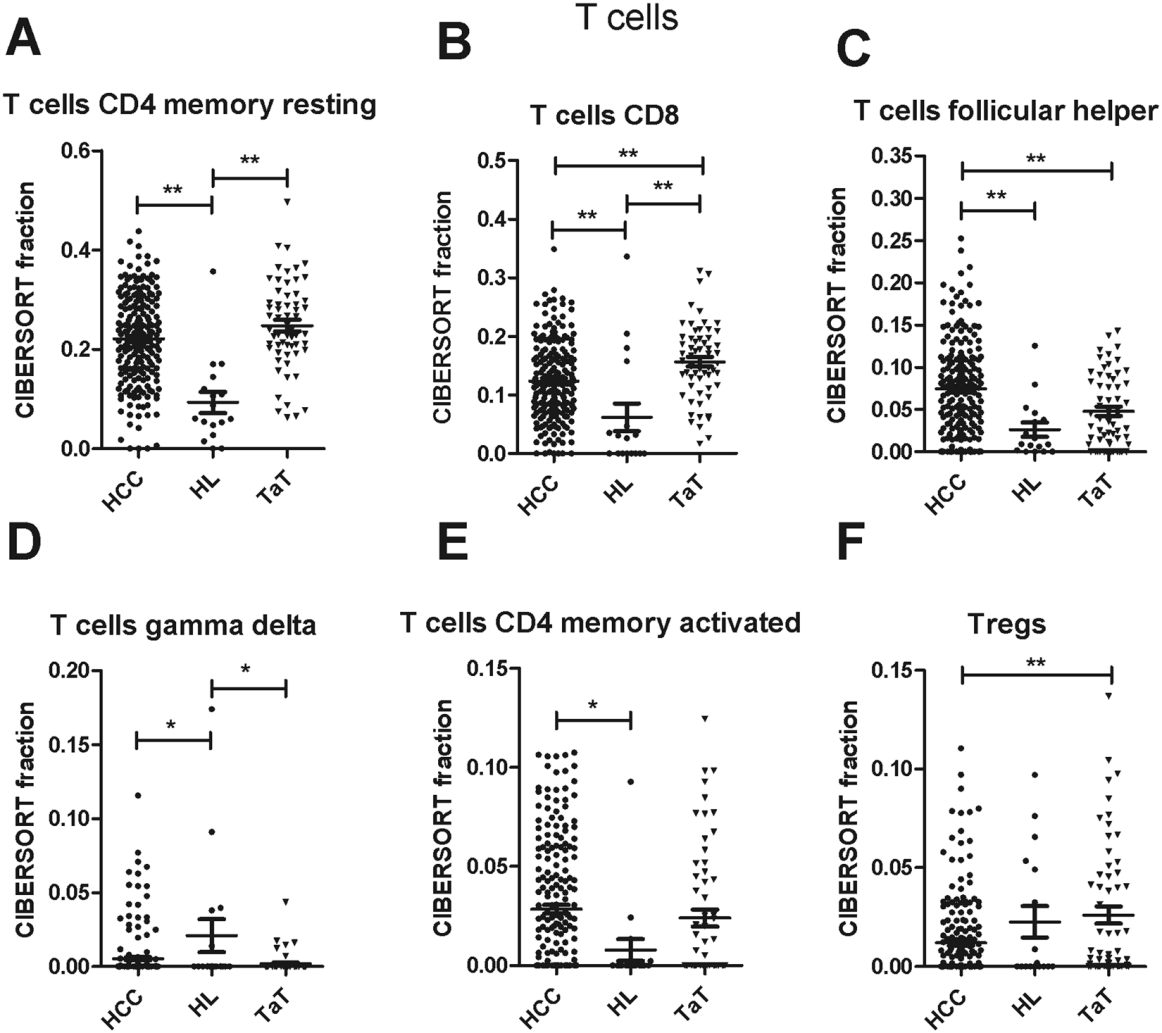
T cell subfractions in human HCC tumor tissue (HCC), adjacent tissue (TaT) and healthy liver. (HL). CIBERSORT immune cell fractions were determined for each patient; each dot represents one patient. Mean values and standard deviations for each cell subset including CD4 memory resting cells (**A**), CD8 cells (**B**), follicular helper (**C**), T cells gamma delta (**D**), CD4 memory activated (**E**) and Tregs (**F**) were calculated for each patient group and compared using one-way ANOVA. *p < 0.05; **p < 0.01.

The three main T cell subpopulations in tissues were CD4^+^ memory resting T cells, CD8^+^ T cells and follicular helper T cells. They were increased in HCC and TaT when compared to healthy liver (Fig. 2A–C, Table 1). Moreover, a small fraction of CD4^+^ memory activated T cells was also increased in HCC and TaT (Fig. 2E). In contrast, gamma delta T cells and regulatory T cells were decreased in HCC when compared to healthy liver (Fig. 2D,F, Table 1). CD8^+^ T cells and Tregs were more frequent whereas follicular helper T cells were less frequent in TaT than in tumor tissues (Fig. 2B,C,F).

### Innate immune cells in HCC

**Figure 3:**
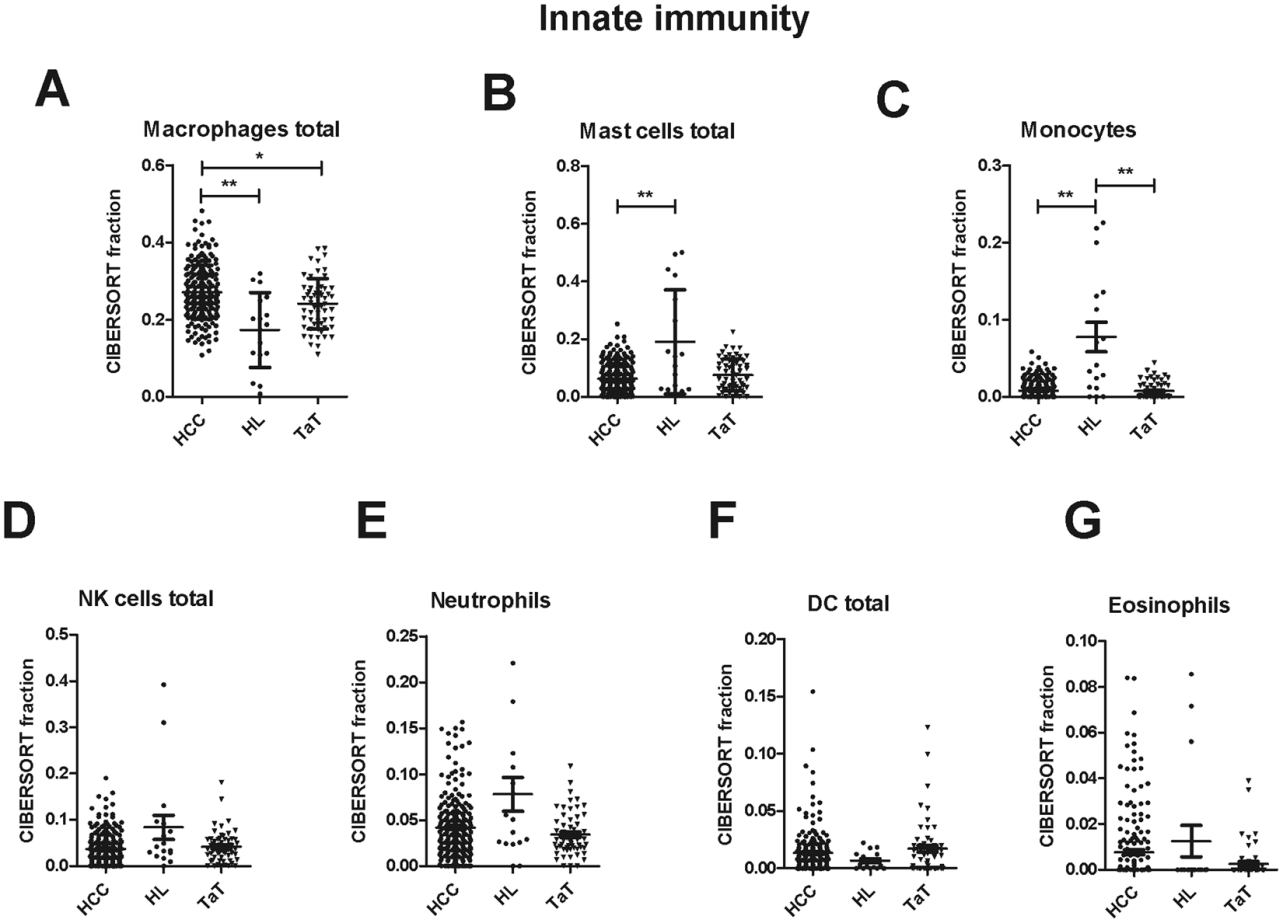
Innate immune response cells in human HCC tumor tissue (HCC), adjacent tissue (TaT) and healthy liver (HL). CIBERSORT immune cell fractions were determined for each patient; each dot represents one patient. Mean values and standard deviations for each cell subset including total macrophages (**A**), total mast cells (**B**), monocytes (**C**), total NK cells (**D**), neutrophils (**E**), total dendritic cells DC (**F**) and eosinophils (**G**) were calculated for each patient group and compared using one-way ANOVA. *p < 0.05; **p < 0.01.

The fraction of macrophages was higher in HCC than in healthy liver and TaT (Fig. 3A). In contrast, monocytes and total mast cells were decreased in HCC (Fig. 3B,C). Fractions of total natural killer (NK) cells, neutrophils, total dendritic cells and eosinophils were not significantly altered among tissues (Fig. 3D–G). Subpopulation analysis revealed that resting dendritic cells (DC) were increased in TaT, whereas activated DC, activated NK and resting NK fractions did not differ (Supplementary Figure 1).

**Figure 4:**
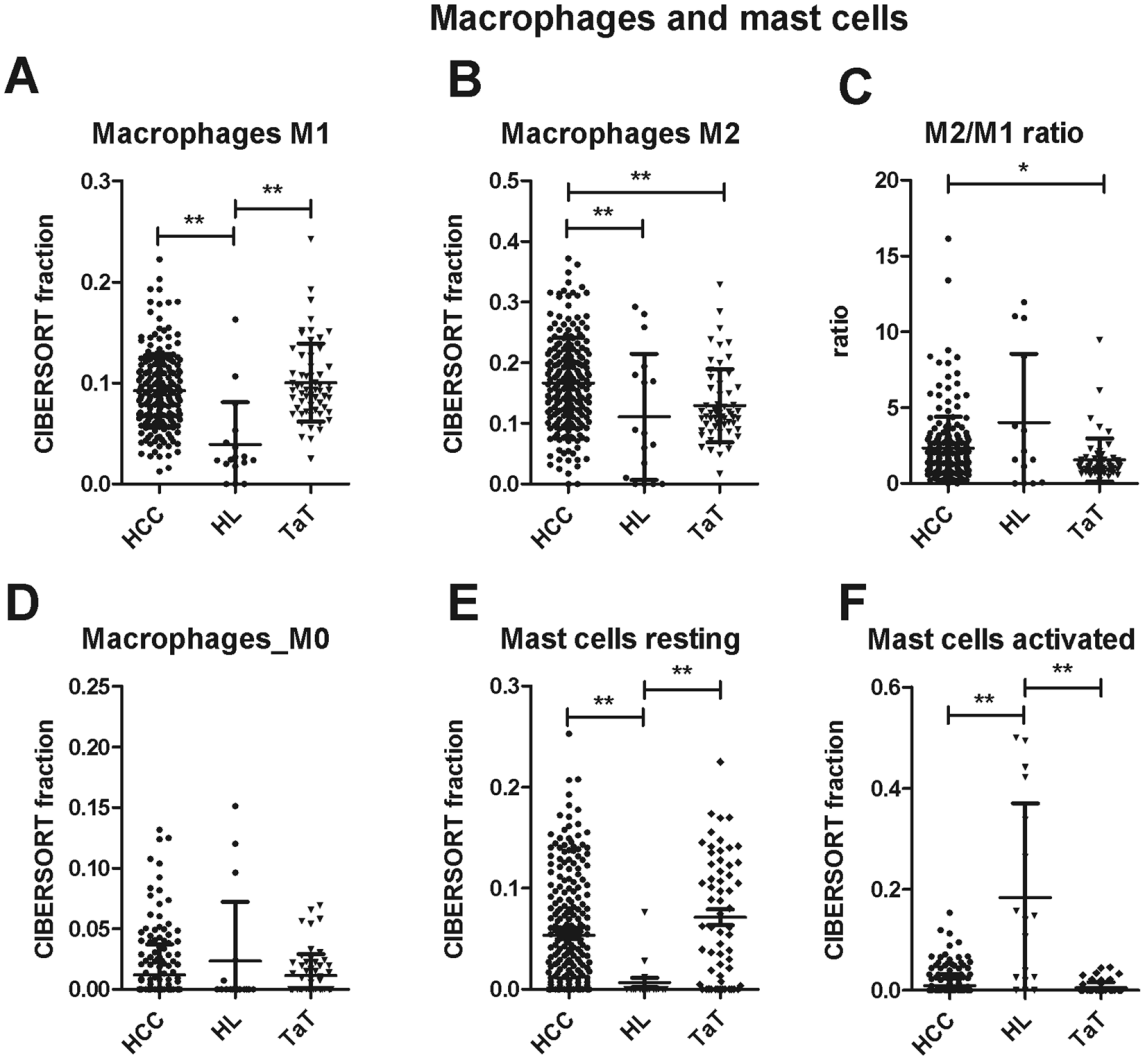
Macrophage and mast cell subfractions in human HCC tumor tissue (HCC), adjacent tissue (TaT) and healthy liver (HL). CIBERSORT immune cell fractions were determined for each patient; each dot represents one patient. Mean values and standard deviations for each cell subset including M1 macrophages (**A**), M2 macrophages (**B**), M2/M1 ratio (**C**), M0 macrophages (**D**), resting mast cells (**E**) and activated mast cells (**F**) were calculated for each patient group and compared using one-way ANOVA. *p < 0.05; **p < 0.01.

M1 macrophages comprised 8.9 ± 3.5% (p < 0.001, n = 198) of total immune cells in HCC. M1 fraction was higher in HCC and TaT than in healthy liver (Fig. 4A). Immune-suppressive, proangiogenic M2 macrophages were specifically enriched in HCC (17.1 ± 7.3%, n = 198, vs 11.0 ± 10.3%, n = 16, in normal tissue, p < 0.001) but not in TaT (Fig. 4B). Correspondingly, the M2/M1 macrophage ratio was higher in HCC than in TaT (Fig. 4C). M0 macrophages comprised 0.9 ± 2.1% (p < 0.001, n = 198) of total immune cells in HCC and were comparable between HCC, TaT and healthy liver (Fig. 4D). Resting mast cells were strongly increased in HCC and TaT when compared to healthy liver, whereas activated mast cells were decreased (Fig. 4E,F).

Alternative algorithms are available for immune cell quantification. We applied two of them, xCell^16^ and EPIC^17^, in order to compare the results for those immune cells types which significantly differed between HCC and TaT. The results are shown in Supplementary Table 1 and Supplementary Table 2. EPIC allows deconvolution of fewer cell types as compared to CIBERSORT, so that only some correlations could be calculated. Moreover, the estimated fractions are referred to the total cell mixture and not only to the total immune cells, as in CIBERSORT results. However, data for B cells, CD8^+^ T cells, macrophages and NK cells calculated by EPIC all correlated with CIBERSORT results (Supplementary Table 1). Similarly, xCell algorithm obtained abundance scores which were mostly in qualitative accordance with CIBERSORT deconvolution results (Supplementary Table 2).

To further elucidate the role of mast cell activation in the HCC immune cell network, we analyzed correlations of resting and activated mast cells with other immune cell populations by calculating r^2^ Pearson correlation coefficients (Supplementary Figure 2). Activated mast cells correlated positively with activated dendritic cells and eosinophils in healthy liver, HCC and TaT. They also correlated positively with other immune cell types of adaptive and innate immune responses in HCC. However, they correlated negatively with plasma cells, Tregs and T follicular helper cells in healthy liver but not in HCC and TaT. Furthermore, activated mast cells correlated positively with gamma delta T cells and naïve B cells in TaT. Resting mast cells correlated positively only with resting NK cells in healthy liver but this correlation was abolished in HCC and TaT. Instead, HCC and TaT showed a correlation between resting mast cells and M0 macrophages (Supplementary Figure 2).

### Immune cell patterns in molecular HCC subclasses

Molecular classification of human HCC led to separation of S1, S2 and S3 subclasses, which display activation of specific signaling pathways and different prognoses^18^. Whereas S1 and S2 exhibit early recurrence and poor prognosis, S3 tumors are well differentiated and show favorable prognosis^18^. Therefore, we investigated differences in immune cell patterns among HCC subclasses. S3 tumors exhibited increased total mast cells when compared to S1 as well as increased M1 macrophages and memory B cells when compared to S1 and S2 tumors (Table 2). Other innate and adaptive immunity cell fractions were similar between subclasses (Table 2). Thus, different molecular HCC subclasses were associated with distinct immune phenotypes. Viral status (HCV, HBV or negative) had no impact on the immune cell composition except for activated mast cells, which were decreased in HCV and HBV infected patients (Supplementary Table 3).

**Table 2:** Comparison of immune cell fractions in percent between three molecular HCC subclasses.

Immune cell type	CIBERSORT fraction in % of all infiltrating immune cells, mean ± SEM			ANOVA p-value (with Bonferroni correction)
	Subclass S1 (n = 19)	Subclass S2 (n = 15)	Subclass S3 (n = 34)	
T cells total	45.58 ± 1.20	42.18 ± 2.21	40.47 ± 1.82	
T cells CD8^+^	12.4 ± 1.70	9.82 ± 2.57	10.69 ± 1.03	0.534
T cells CD4^+^ memory resting	21.8 ± 2.02	24.2 ± 2.86	22.98 ± 1.48	0.975
**T cells CD4** ^**+**^ **memory activated**	**2.87 **±** 0.66**^**a**^	**2.31 **±** 0.79**	**1.09 **±** 0.33**^**a**^	**0.03**
T cells Follicular Helper	5.46 ± 1.19	5.05 ± 1.35	3.81 ± 0.66	0.321
Tregs	3.00 ± 0.79	0.81 ± 0.46	1.91 ± 0.49	0.191
B cells total	6.39 ± 0.93	5.90 ± 1.19	4.82 ± 0.49	
B cells memory	2.26 ± 0.73	2.73 ± 1.68	5.22 ± 0.16	0.065
B cells naïve	4.10 ± 0.99	5.63 ± 1.22	4.31 ± 0.53	0.663
Macrophages total	28.14 ± 1.97	27.24 ± 1.41	28.81 ± 1.41	
M0 macrophages	3.30 ± 0.92	2.28 ± 0.86	2.35 ± 0.55	0.326
**M1 macrophages**	**9.32 **±** 0.72**^**b**^	**9.22 **±** 0.81**^**c**^	**12.55 **±** 0.72**^**b,c**^	**0.003**
M2 macrophages	15.51 ± 2.04	15.75 ± 1.70	13.91 ± 1.36	0.452
M1/M2 ratio	0.98 ± 0.22	0.73 ± 0.11	1.16 ± 0.17	
Mast cells	6.41 ± 1.17	9.66 ± 1.47	10.49 ± 1.06	0.115
Neutrophils	4.71 ± 0.64	4.92 ± 1.17	5.49 ± 0.62	0.753
Dendritic cells	2.17 ± 0.84	1.70 ± 0.43	2.04 ± 0.39	0.951
Monocytes	0.21 ± 0.15	0.62 ± 0.21	0.48 ± 0.17	0.485
Eosinophils	1.91 ± 0.13	0	0	0.091

^a^Different at p = 0.037.

^b^Different at p = 0.011.

^c^Different at p = 0.018, ANOVA with Bonferroni correction.

**Figure 5:**
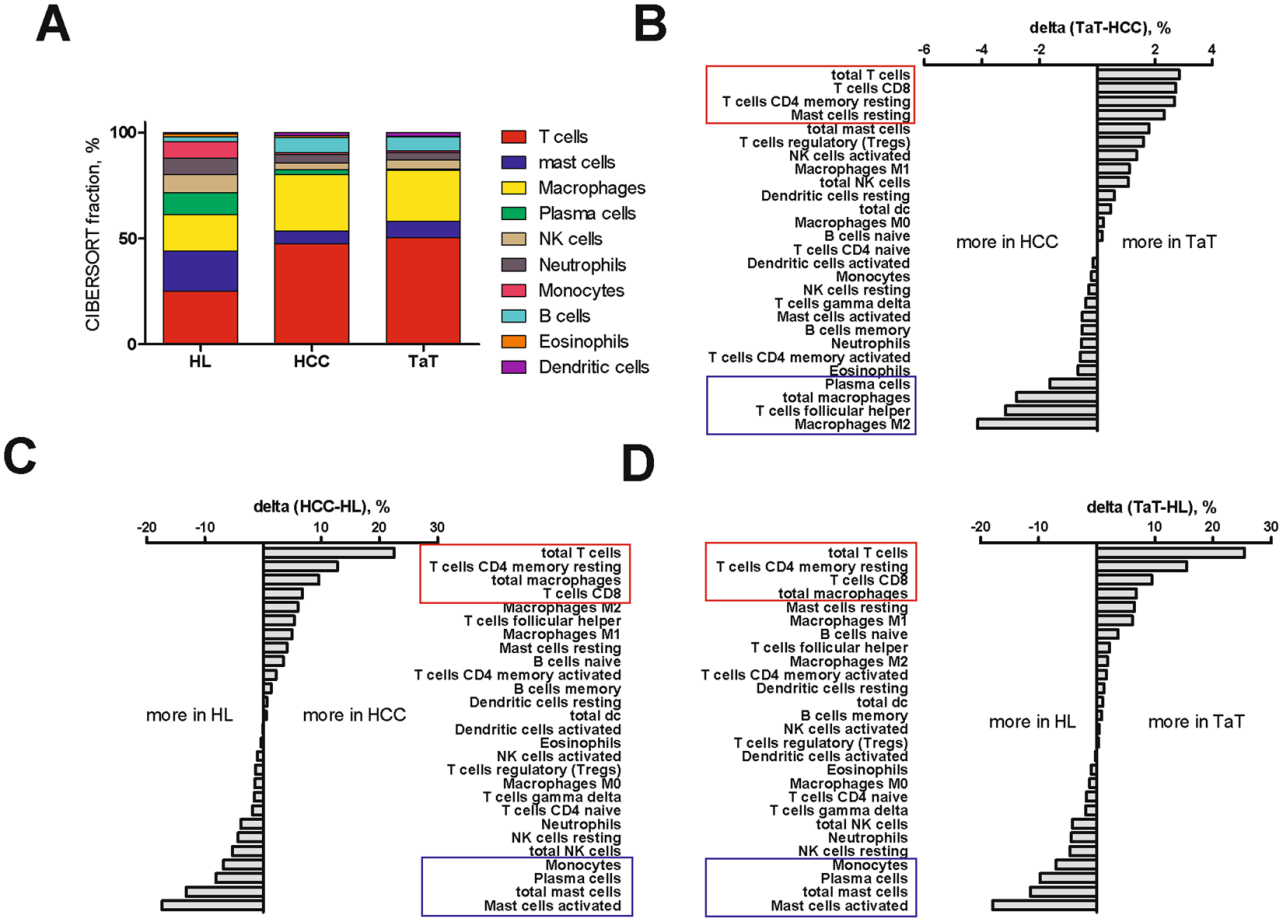
Immune cell composition in HCC tumor (HCC), adjacent tissues (TaT) and healthy livers (HL). (**A**) Composition of infiltrating immune cells in HCC, TaT and HL summarized from calculated mean values for each patient group. (**B**–**D**) Quantified changes of infiltrating immune cell composition between TaT and HCC (**B**), HL and HCC healthy liver and tumor tissue (**C**) and between HL and TaT (**D**).

The immune cell composition in HCC and TaT differed substantially from that of healthy liver tissue (Fig. 5A–D). In particular, T cells (25.0 ± 8.6%), mast cells (19.0 ± 18.1%) and macrophages (17.3 ± 9.7%) were most frequent in healthy liver (n = 16) and prevailed over NK cells (8.4 ± 10.7, p = 0.04), monocytes (7.8 ± 7.9%, p = 0.008) and neutrophils (7.8 ± 7.6%, p = 0.02). In HCC and TaT, almost 50% of total immune cells were T cells. Macrophages were more frequent than mast cells (Fig. 5A–D). Activated mast cells were barely found in HCC but mainly in healthy liver (Fig. 5B,C). Importantly, higher relative proportion of resting mast cells in HCC showed a trend toward shorter survival of patients (p = 0.13, data not shown)

### Total Immune cell infiltration and its correlation with immune cell types

The extent of immune cell infiltration into tumors has important prognostic value in HCC and other cancers^5,9,15,19^. Therefore, we used the p-value of CIBERSORT deconvolution as a surrogate parameter for the magnitude of total immune cell infiltration as lower p-values are associated with higher total infiltration^13,15^ and assessed correlations with immune cell types. Indeed, CIBERSORT p-value correlated with a new CIBERSORT feature “Absolute Score”. The “Absolute Score” is estimated as the median expression level of all genes in the signature matrix divided by the median expression level of all genes in the mixture. This score is used by the CIBERSORT “absolute mode” (currently under development) to scale the relative cell fractions to absolute abundances (https://cibersort.stanford.edu). As expected, we found that CIBERSORT p-values inversely correlated with the “Absolute Score” (Spearman-Rho correlation coefficient r^2^ = −0.639, p = 6e-51, n = 432).

**Figure 6:**
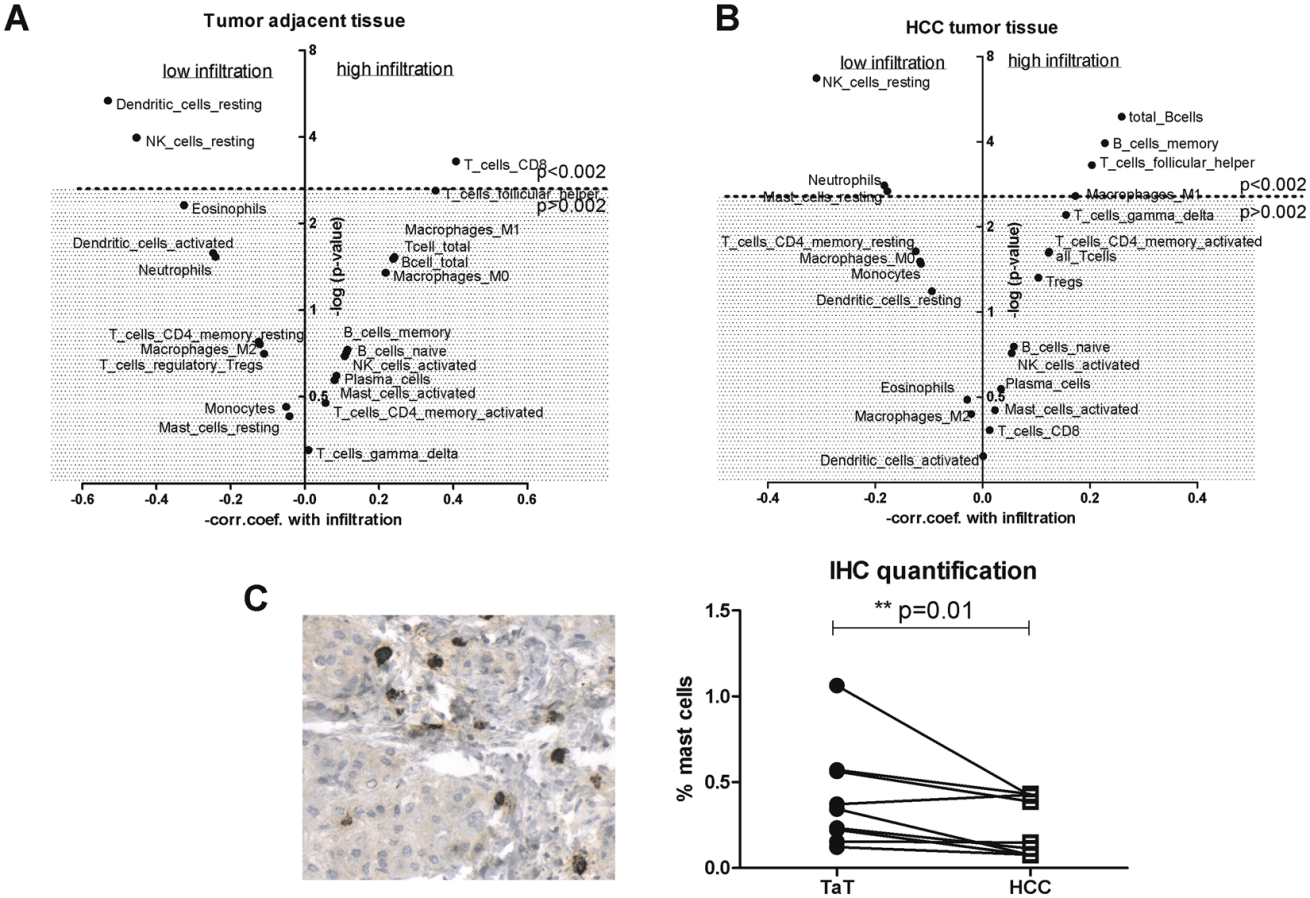
Correlations of immune cells with CIBERSORT p-values in HCC tumor and adjacent tissues. Impact of individual immune cell subsets on the total immune cell infiltration within TaT (**A**) and HCC (**B**). The dotted line represents p = 0.002 boundary (Bonferroni correction), all the cell subsets above this line are significantly associated with total immune infiltration with p-values < 0.002. The X-axis shows Pearson correlation coefficients between cell subset and CIBERSORT p-values; positive values indicate an infiltration increase with increased cell subset, whereas negative values indicate an infiltration decrease. (**C**) mast cell tryptase staining in human HCC tissue and the summary of immunohistochemical evaluation in ten human HCC tumor tissues (HCC) and corresponding tumor adjacent tissues (TaT). Mast cell density was calculated across the slide by tissue morphometric analysis and expressed as percent of total cells. Two –tailed p-value p = 0.0098, Wilcoxon singed rank test.

The degree of immune cell infiltration into the tumor and surrounding tissue is an important prognostic factor. To characterize the interdependence between immune cell composition and the degree of immune cell infiltration in HCC, we calculated the correlations of 22 immune cell types with CIBERSORT p-values. Our results revealed that CD8^+^ T cells are mainly associated with high immune cell infiltration into TaT (Fig. 6A). In HCC, high immune cell infiltration was mainly linked with the presence of total B cells, memory B cells, follicular helper T cells and M1 macrophages (Fig. 6B). On the contrary, lower immune cell infiltration in HCC was rather associated with the presence of neutrophils, resting NK cells and resting mast cells (Fig. 6B).

To further explore mast cells abundance in HCC, we used the “absolute mode” of CIBERSORT to quantify the abundances of resting, activated and total mast cells in HCC and TaT (Supplementary Table 5). Absolute values for total and resting mast cells were significantly diminished in HCC tumor tissue as compared to tumor adjacent tissue (Supplementary Table 5).

In order to verify the explorative data obtained for mast cells, we evaluated mast cell density by immunohistochemistry in ten human HCC tumor tissues and ten corresponding adjacent tissues. Examples of mast cell tryptase staining in HCC tissue together and quantification summary are shown in Fig. 6C. In agreement with CIBERSORT results (Supplementary Table 5), mast cell density was reduced in HCC as compared to tumor adjacent tissue.

## Discussion

In this study, we applied CIBERSORT to assess differential immune cell infiltration in healthy human liver, HCC and HCC adjacent tissue.

We observed considerable differences in immune cell composition between HCC and healthy liver whereas molecular HCC subclasses displayed only subtle differences. However, S3 tumors showed an enrichment of M1 macrophages which can be tumor-suppressive and might contribute to the favorable prognosis of this HCC subclass^20^.

To our knowledge, the present study shows for the first time that the mast cells in HCC are largely inactive. Since mast cell activation by IgE is supposed to protect from cancer^21^, inactivation of mast cells in HCC may facilitate immune escape and thus favor tumor growth.

Although different stimuli can activate mast cells^22^, CIBERSORT enumerates specifically IgE activated mast cells because the gene expression signature used for deconvolution was obtained from mast cells stimulated by IgE^13^.

Mast cells are key regulators of immune effector cells^23^. Therefore, their activation could be a desired aim of immunotherapy. Mast cells are attractive targets as they are abundant and immobile in the liver and in tumors, relatively radioresistant and more resistant to chemotherapeutics than other rapidly dividing immune cells^22^.

The mechanisms behind mast cell inactivation in HCC remain unknown. Mast cell activator IgE has been detected in HCC, at least in patients with HBV-associated HCC^24^, and seems not to be a limiting factor. However, tumor cells might release certain metabolites that potentially inhibit mast cell activation. We hypothesize that tumor cell-derived metabolites such as oxidized natural polyamines might be responsible for mast cell inhibition in HCC. Indeed, natural polyamines spermine and spermidine, when oxidized by polyamine oxidase, prevented mast cell activation by IgE *in vitro*^25^. Malignant cells contain high concentrations of polyamines^26^ and polyamine oxidase is highly expressed in the liver^27^ thus supporting the relevance of polyamine oxidation for HCC. In line, polyamine oxidase inhibitor delayed experimental tumor growth^28^.

When compared to EPIC and xCell, CIBERSORT is the only algorithm that allows discrimination between resting and IgE activated mast cells. CIBERSORT calculations were confirmed by immunohistochemical mast cell quantification in tumor and adjacent tissues of Austrian HCC patients, a small but completely independent cohort from that used for calculations. Our novel findings on mast cells in HCC provoke more detailed future studies to assess the potential of mast cell activation in HCC immunotherapy.

It has been previously reported, that T and B cells are present in immune cell infiltrates of HCC and that the degree of tumor infiltrating T and B cells correlates with improved survival of HCC patients^19^. Our data are in agreement with these findings. They also reveal that total B cells and – to a lesser extend - total T cells are significant contributors to the total immune infiltration into HCC tumors (Fig. 6B). Moreover, we identified the involved T and B cell subsets as T follicular helper cells and memory B cells and provide additional important information on the immune cell composition of HCC adjacent tissues.

The prognostic importance of immune cell infiltration has been recognized for different solid tumor types. For example in colon cancer, the so called immunoscore - which reflects the type, number and distribution of immune cells into the tumor - has been introduced and shows prognostic value^9^. Recent application of the immunoscore in HCC revealed that increased intratumoral densities of CD3^+^ and CD8^+^ cells were linked to prolonged survival^29,30^. Interestingly, immunotherapy can modify infiltration of cytotoxic CD8^+^ T cells^31^. We could confirm the presence of CD8^+^ T cells in HCC tumors. However, tumor adjacent tissue showed even higher CD8^+^ T cell frequency (Fig. 2B) possibly indicating an impeded infiltration into the tumor.

Whereas surgical resection of human tumors provides tumor tissue and tumor adjacent tissue for research purposes (only if ethical issues are properly considered), the access to liver samples and datasets from healthy humans is much more limited. Healthy liver samples are rare and are mostly collected after a sudden death or at liver transplantation setting, which potentially influences immune infiltration. In addition, the degree of immune infiltration into the healthy liver seems to be lower than in liver cancer. In line, for more than the half of datasets from healthy livers (25 of 41), we did not obtain statistical significance of the deconvolution results (i.e. p < 0.05), probably because of unfavorable signal/noise ratio. However, the most differences between immune cell types remained valid even if less samples only from persons with sudden death were included (not shown).

In summary, we demonstrate that deconvolution of whole tissue gene expression data by CIBERSORT provides refined information on the immune cell landscape of HCC. We show that the presence of resting or activated mast cells is indicative for the presence of other immune cell types and might be relevant for HCC patient prognosis. Deviations of the HCC immunoprofile from healthy liver may become a valuable tool to identify novel targets for immunotherapies and to individualize treatment strategies in patients with HCC.

## Materials and Methods

CIBERSORT is an analytical tool which accurately quantifies the relative levels of distinct immune cell types within a complex gene expression mixture (https://cibersort.stanford.edu)^13^. To characterize and to quantify each immune cell subtype, CIBERSORT uses gene expression signatures consistent of ~500 genes. Here, we applied the original CIBERSORT gene signature file LM22 which defines 22 immune cell subtypes and analyzed datasets from human hepatocellular carcinoma (HCC), HCC tumor adjacent tissue (TaT) and healthy livers (HL). Public available gene expression profiles from human normal tumor-free livers (HL, n = 41), HCC tumors (HCC, n = 305) and HCC tumor adjacent tissues (TaT, n = 82). All GEO numbers are given in Table 3. The data are normalized using the cubic spline algorithm. All samples were analysed for immune cell profiles by CIBERSORT, the number of permutations being set to 100^13^. 22 immune cell types together with CIBERSORT metrics as Pearson correlation coefficient, CIBERSORT p-value and root mean squared error (RMSE) were quantified for each sample. CIBERSORT p-value reflects the statistical significance of the deconvolution results across all cell subsets and is useful for filtering out deconvolution with less significant fitting accuracy (https://cibersort.stanford.edu). From all the samples analyzed, we have selected 16/198/60 HL/HCC/TaT samples respectively which met the requirements of CIBERSORT p-value ≤ 0.05. The complete list of the selected samples is given in Table 3. Immune cell profile was calculated for each sample and mean values for each tissue type (HL, HCC and TaT) were calculated. One-way- ANOVA was applied to analyze the differences between healthy livers, HCC tumors and adjacent tissues. For resting and activated mast cells, Pearson correlation coefficients with other immune cells types were calculated using SPSS 24.0 software.

**Table 3:** List of datasets used for estimation of immune cell profiles.

Tissues	Datasets used for CIBERSORT analysis	References
Tumor tissues, HCC (n = 198)	GSM256426, GSM256432, GSM256445, GSM256476, GSM256483, GSM256504, GSM256507, GSM256524, GSM256549, GSM256593, GSM256598, GSM256633, GSM256645, GSM256650, GSM256657, GSM256663, GSM256677, GSM256686, GSM256688, GSM256703, GSM256721, GSM256726, GSM256439, GSM256442, GSM256480, GSM256588, GSM256616, GSM256702, GSM256722, GSM256434, GSM256468, GSM256471, GSM256496, GSM256539, GSM256546, GSM256558, GSM256565, GSM256570, GSM256576, GSM256584, GSM256595, GSM256596, GSM256625, GSM256644, GSM256659, GSM256665, GSM256672, GSM256679, GSM256680, GSM256681, GSM256693, GSM256495, GSM256556, GSM256590, GSM256651, GSM256697, GSM256701, GSM256428, GSM256430, GSM256443, GSM256457, GSM256459, GSM256460, GSM256462, GSM256463, GSM256464, GSM256465, GSM256467, GSM256469, GSM256470, GSM256472, GSM256477, GSM256481, GSM256482, GSM256484, GSM256485, GSM256486, GSM256488, GSM256492, GSM256499, GSM256505, GSM256506, GSM256508, GSM256509, GSM256510, GSM256511, GSM256512, GSM256513, GSM256514, GSM256515, GSM256517, GSM256533, GSM256534, GSM256538, GSM256540, GSM256542, GSM256543, GSM256547, GSM256551, GSM256553, GSM256555, GSM256557, GSM256560, GSM256562, GSM256566, GSM256567, GSM256569, GSM256573, GSM256574, GSM256577, GSM256578, GSM256581, GSM256582, GSM256583, GSM256585, GSM256587, GSM256589, GSM256591, GSM256594, GSM256600, GSM256602, GSM256606, GSM256607, GSM256608, GSM256611, GSM256613, GSM256614, GSM256615, GSM256617, GSM256619, GSM256620, GSM256623, GSM256624, GSM256628, GSM256630, GSM256631, GSM256637, GSM256638, GSM256639, GSM256640, GSM256643, GSM256646, GSM256647, GSM256649, GSM256652, GSM256656, GSM256664, GSM256674, GSM256675, GSM256682, GSM256683, GSM256684, GSM256689, GSM256690, GSM256692, GSM256695, GSM256698, GSM256711, GSM256716, GSM256720, GSM256728, GSM256729, GSM256440, GSM256444, GSM256450, GSM256458, GSM256474, GSM256478, GSM256498, GSM256518, GSM256535, GSM256536, GSM256550, GSM256554, GSM256559, GSM256561, GSM256563, GSM256564, GSM256571, GSM256579, GSM256580, GSM256586, GSM256592, GSM256605, GSM256609, GSM256610, GSM256612, GSM256627, GSM256632, GSM256634, GSM256635, GSM256636, GSM256641, GSM256642, GSM256673, GSM256687, GSM256696, GSM256724	^32^
Tumor adjacent tissues, TaT (n = 60)	GSM256354, GSM256362, GSM256377, GSM256408, GSM256409, GSM256410, GSM256415, GSM256418, GSM256367, GSM256374, GSM256350, GSM256355, GSM256360, GSM256368, GSM256375, GSM256380, GSM256381, GSM256394, GSM256401, GSM256411, GSM256412, GSM256416, GSM256419, GSM256342, GSM256349, GSM256364, GSM256369, GSM256371, GSM256379, GSM256399, GSM256404, GSM256405, GSM256406, GSM256407, GSM256413, GSM256420, GSM256423, GSM256344, GSM256351, GSM256358, GSM256359, GSM256366, GSM256372, GSM256376, GSM256378, GSM256383, GSM256384, GSM256385, GSM256386, GSM256387, GSM256388, GSM256389, GSM256390, GSM256393, GSM256395, GSM256396, GSM256397, GSM256400, GSM256402, GSM256417	^32^
Healthy Livers, HL (n = 16)	GSM372247, GSM372248, GSM372249, GSM372599, GSM372600, GSM373314, GSM373315, GSM373324	^33^
	GSM35982	^34^
	E-MTAB-3732_Sample_5242 (GSM155926), E-MTAB-3732_Sample_10761 (GSM155988)	^35,36^
	E-MTAB-3732_Sample_5273 (GSM176332),	^35^
	E-MTAB-3732_Sample_10714 (GSM80730),	^35,37^
	E-MTAB-3732_Sample_1396 (E-AFMX-11HL5), E-MTAB-3732_Sample_8377 (E-AFMX-11HL4),	^35,38^
	E-MTAB-3732_Sample_8656 (GSM319287)	^35,39^

Total macrophage fraction was calculated as a sum of M0, M1 and M2 macrophage fractions. Total T cells were calculated as a sum of CD8+ T cells, CD4+ naïve T cells, CD4+ memory resting T cells, CD4+ memory activated T cells, follicular helper T cells, regulatory T cells (Tregs) and T cells gamma delta fractions.

Log-rank Mantel-Cox test was applied to compare the survival curves between the patient groups using SPSS 24.0 and GpaphPadPrism Software.

To obtain deconvolution of expression data with EPIC^17^, all expression data have been concatenated in a single file and duplicate gene symbols have been resolved by selecting the gene with the highest mean across all samples. Deconvolution was then performed considering the signature matrix defined for tumor data (“TRef”). The Immune Infiltration was estimated by summing up the fractions of: B cells, CD4+ T cells, CD8+ T cells, macrophages, and natural killer (NK) cells. For comparison with CIBERSORT results, only the immune-cell fractions were extracted from EPIC results and re-normalized so to sum up to one. CIBERSORT fractions for naïve B cells and memory B cells were aggregated into B cells, M0, M1, and M0 macrophages into macrophages, and resting and activated NK cells into NK cells. The agreement between EPIC and CIBERSORT results was estimated with Pearson’s correlation.

For computation of abundance scores with xCell^16^, all expression data have been concatenated in a single file and duplicate gene symbols have been resolved by selecting the gene with the highest mean across all samples. Abundance scores were then computed from the expression data with xCell (xCellAnalysis function run with the “rnaseq = FALSE” option). For comparison purposes, CIBERSORT fractions for memory CD4+ T cells, NK cells, and mast cells were computed aggregating the proportions of resting and activated cells.

Mast cells were evaluated immunohistochemically using staining for tryptase. After de-paraffinization, heat-induced epitope retrieval was performed. The slides were cooled down, washed twice with PBS and permeabilized by 0.2% Tween in PBS. Unspecific background was blocked by 5% FCS in PBS for 30 min at room temperature. First antibody mouse anti-human mast cell tryptase (clon AA1, BioRad) was diluted 1:10000 in 5% FCS and incubated overnight. After the washing step, Dako polymer (HRP Mouse Envision Kit, Dako, Agilent, USA) was applied for 30 min at room temperature. DAB (Dako, Agilent, USA) chromogen/substrate were applied for 30 s and the slides were washed with aqua dest. Counterstaining was performed by hematoxylin and tryptase-positive cells were evaluated by tissue morphometric analysis of digitized slides using the Tissue Studio® software (Definiens, Munich, Germany). Slides were digitized using a Pannoramic Midi Slide Scanner (3Dhistech, Budapest, Hungary). HCC tumor tissue and corresponding tumor adjacent tissue from ten patients were evaluated. All the patients had histologically confirmed HCC and underwent orthotopic liver transplantation at Vienna General Hospital, Austria. Clinical data of the patients are summarized in Supplementary Table 6. Data analysis was performed in accordance with guidelines of the local Ethics Committee.

### Data availability

The complete list of analyzed datasets (ArrayExpress) for each group is given in Table 3. The immune profile datasets generated by CIBERSORT for each sample Table 3 during the current study are available from the corresponding author on reasonable request.

## Electronic supplementary material

Supplementary Information
