# Supplementary material for: Deviations of the immune cell landscape between healthy liver and hepatocellular carcinoma
Source: Sci Rep. 2018 Apr 18;8:6220. doi: 10.1038/s41598-018-24437-5 (PMC5906687; doi:10.1038/s41598-018-24437-5)
Supplement: Supplementary file 1 — Supplementary Information [file 41598_2018_24437_MOESM1_ESM.pdf]

Supplementary information to the manuscript:

Deviations of the immune cell landscape between healthy liver and hepatocellular carcinoma

Nataliya Rohr-Udilova[1*], Florian Klinglmüller[2], Rolf Schulte-Hermann[3], Judith Stift[4], Merima Herac[4], Martina Salzmann[5], Francesca Finotello[6], Gerald Timelthaler[4], Georg Oberhuber[3], Matthias Pinter[1], Thomas Reiberger[1], Erika Jensen-Jarolim[5,7], Robert Eferl[4], Michael Trauner[1]

[1]Division of Gastroenterology and Hepatology, Department of Internal Medicine III, Medical University of Vienna, Waehringer Guertel 18-20, A-1090 Vienna, Austria; nataliya.rohr-udilova@meduniwien.ac.at

[2] Centre for Medical Statistics, Informatics and Intelligent Systems, Medical University of Vienna, Spitalgasse 23, A-1090 Vienna Austria

[3]Institute of Cancer Research, Internal Medicine I, Medical University of Vienna and Comprehensive Cancer Center (CCC), Borschkegasse 8a, A-1090 Vienna, Austria

[4] Clinical Institute of Pathology, Medical University of Vienna, Waehringer Guertel 18-20, A-1090 Vienna, Austria

[5]Institute of Pathophysiology and Allergy Research, Center of Pathophysiology, Infectiology and Immunology, Medical University of Vienna, Vienna, Austria

[6] Division of Bioinformatics, Biocenter, Medical University of Innsbruck, Innrain 80-82, A-6020 Innsbruck, Austria

[7]Comparative Medicine, The Interuniversity Messerli Research Institute of the University of Veterinary Medicine Vienna, Medical University Vienna and University Vienna, Vienna, Austria

[*] *Correspondence to:* Dr. Nataliya Rohr-Udilova

Division of Gastroenterology and Hepatology,

Department of Internal Medicine III,

Medical University of Vienna

Waehringer Guertel 18-20

A-1090 Vienna, Austria

Tel.: +43 1 40400 47440

Fax: + 43 1 40400 47350

e-mail: nataliya.rohr-udilova@meduniwien.ac.at

Supplementary Table 1. Pearson's correlation of CIBERSORT and EPIC deconvolution results.

Cell type	Correlation	p-value
**B cells**	0.52	<2e-16
**Macrophages**	0.22	1.60e-05
**NK cells**	0.23	3.70e-06
**$CD8^+$ T cells**	0.21	3.60e-05

Supplementary Table 2. Comparison of immune cell abundance in HCC calculated by xCell and CIBERSORT 3algorithms.

Cell type /parameter	xCell score, mean+- SD			Pearson correlation between xCell and CIBERSORT		Qualitative consistency xCell vs CIBERSORT
	HCC tissue	Tumor adjacent tissue (TaT)	p-value (HCC vs TaT)	correlation coefficient	p-value	
Immunoscore	0.42+0.10	0.54+0.09	$4 \cdot 10^{-20}$	n.d.	n.d.	(yes)
Plasma cells	0.025+0.026	0.0047+0.011	10^{-11}	0.42	$<10^{-16}$	yes
T cells CD8+	0.091+0.048	0.11+0.06	$5 \cdot 10^{-5}$	0.53	$<10^{-16}$	yes
Tregs	0.16+ 0.06	0.14+ 0.06	$8 \cdot 10^{-3}$	-0.19	0.00013	no
Total Macrophages	0.038+0.028	0.012+0.015	$2 \cdot 10^{-14}$	n.d.	n.d.	yes
M1 macrophages	0.0023+0.0049	0.00034+0.00111	$4 \cdot 10^{-4}$	0.02	0.7	(yes)
M2 macrophages	0.11+0.03	0.096+0.039	10^{-3}	0.29	$4 \cdot 10^{-9}$	yes

Supplementary Table 3. Impact of the viral status in HCC patients on CIBERSORT immune cell frequencies.

CIBERSORT results	Viral status						ANOVA p-values (between the groups)
	negative (n=3)		HCV (n=57)		HBV (n=17)		
	mean	stdev	mean	stdev	mean	stdev	
B_cells_naive	0,026	0,028	0,048	0,039	0,040	0,041	0,539
B_cells_memory	0,011	0,018	0,009	0,020	0,012	0,019	0,859
Plasma_cells	0,006	0,010	0,006	0,025	0,008	0,025	0,980
T_cells_CD8	0,099	0,022	0,117	0,081	0,091	0,056	0,442
T_cells_CD4_memory_resting	0,247	0,047	0,222	0,106	0,237	0,052	0,793
T_cells_CD4_memory_activated	0,028	0,016	0,023	0,030	0,012	0,020	0,315
T_cells_follicular_helper	0,015	0,013	0,047	0,046	0,039	0,051	0,472
Tregs	0,027	0,046	0,020	0,029	0,017	0,028	0,850
T_cells_gamma_delta	0,000	0,000	0,001	0,005	0,000	0,000	0,843
NK_cells_resting	0,035	0,037	0,018	0,035	0,026	0,038	0,570
NK_cells_activated	0,040	0,042	0,044	0,043	0,029	0,033	0,443
Monocytes	0,000	0,000	0,005	0,010	0,003	0,008	0,609
Macrophages_M0	0,048	0,035	0,029	0,039	0,022	0,030	0,514
Macrophages_M1	0,072	0,034	0,110	0,040	0,105	0,033	0,244
Macrophages_M2	0,126	0,043	0,145	0,084	0,157	0,068	0,787
Dendritic_cells_resting	0,022	0,027	0,022	0,030	0,034	0,035	0,422
Dendritic_cells_activated	0,000	0,000	0,000	0,001	0,000	0,000	0,843
Mast_cells_resting	0,095	0,082	0,080	0,059	0,107	0,071	0,280
**Mast_cells_activated**	**0,051*#**	**0,089**	**0,002***	**0,016**	**0,005#**	**0,016**	**0,001**
Eosinophils	0,000	0,000	0,001	0,004	0,000	0,000	0,734
Neutrophils	0,052	0,029	0,052	0,035	0,057	0,037	0,882
P_value	0,173	0,146	0,122	0,174	0,142	0,228	0,844
total_Tcells	0,514	0,051	0,472	0,078	0,450	0,085	0,363
total_Bcells	0,037	0,025	0,056	0,036	0,052	0,039	0,617
total_NK	0,075	0,009	0,062	0,042	0,055	0,034	0,695
total_Mast_cells	0,146	0,008	0,082	0,058	0,112	0,065	0,051
total_MF	0,247	0,038	0,284	0,084	0,283	0,058	0,718
total_DC	0,022	0,027	0,023	0,030	0,034	0,035	0,429
M1_M2_ratio	0,716	0,633	1,033	0,946	0,840	0,491	0,625

Viral status was available for a subset of 78 patients. Of these, n= 57 were HCV positive, n=17 were HBV positive, n=3 were HBV/HCV negative and n=1 was positive for both HBV/HCV . Double positive patient was excluded from analysis. Means and standard deviations were calculated for each group.
* p=0.001 vs HCV after Bonferroni correction; # p=0.003 vs HBV after Bonferroni correction

Supplementary Table 4. Sample characteristics of healthy liver datasets.

Dataset	Sex	Age, years	Condition	Reference
**GSM372247**	female	27	Healthy adult, sudden death	33
**GSM372248**	female	29	Healthy adult, sudden death	33
**GSM372249**	male	29	Healthy adult, sudden death	33
**GSM372599**	female	48	Liver transplant	33
**GSM372600**	male	34	Liver transplant	33
**GSM373314**	male	53	Liver transplant	33
**GSM373315**	female	48	Liver transplant	33
**GSM373324**	female	47	Liver transplant	33
**GSM35982**	NA	NA	Healthy liver	34
**GSM155926**	NA	NA	Healthy liver	35,36
**GSM155988**	NA	NA	Normal liver tissue	35,36
**GSM176332**	male	NA	Normal liver tissue	35
**GSM80730**	female	39	Healthy adult, sudden death	35,37
**GSM319287**	NA	NA	Liver transplant	35,39
**E-AFMX-11HL4**	male	29	Normal liver tissue	35,38
**E-AFMX-11HL5**	Male	NA	Normal liver tissue	35,38

Supplementary Table 5. Estimated absolute mast cell infiltration in HCC and TaT using “absolute score” feature of CIBERSORT.

	HCC (n=183)	TaT (n=65)	p-value for means comparison by ANOVA
Mast cells resting	0,029+0,031*	0,046+0,039*	0,004
Mast cell activated	0,0063+0,014	0,0031+0,0077	0,522
Mast cell total	0,035+0,028#	0,049+0,035#	0,028

Only the datasets with p<0.05 of CIBERSORT calculations in “absolute score” mode were selected for analysis.

#, * the corresponding TaT and HCC values differ significantly at p=0.05.

Supplementary Table 6. Clinical characteristics of HCC patients used for immunohistochemical quantitation of mast cells in tumor and tumor adjacent tissues shown in Fig. 6C.

	sex	Age	AFP	tumor size (cm)	tumor-grading	underlying disease	pM	pN	vascular invasion
**Patient1**	male	65	n.a.	10	3	HCV	0	N0	1
**Patient2**	male	49	2,9	1,1	1	HCV	0	N0	0
**Patient3**	male	55	n.a.	6	2	HCV	n.a.	n.a.	0
**Patient4**	female	68	7,8	6	2	HCV	0	N0	0
**Patient5**	male	55	4,4	1,1	2	HCV	0	N0	0
**Patient6**	male	61	641	0,5	2	HCV	0	N0	0
**Patient7**	male	50	85	0,5	2	HBV+HCV	0	N0	0
**Patient8**	male	58	38,9	0,6	1	HCV	0	N0	0
**Patient9**	male	58	22	2,2	2	HCV	0	N0	0
**Patient10**	male	65	8,2	2,4	2	HCV	0	N0	0
**mean**		58	101	3,0					
**stdev**		6	220	3,2					

Supplementary Figure 1

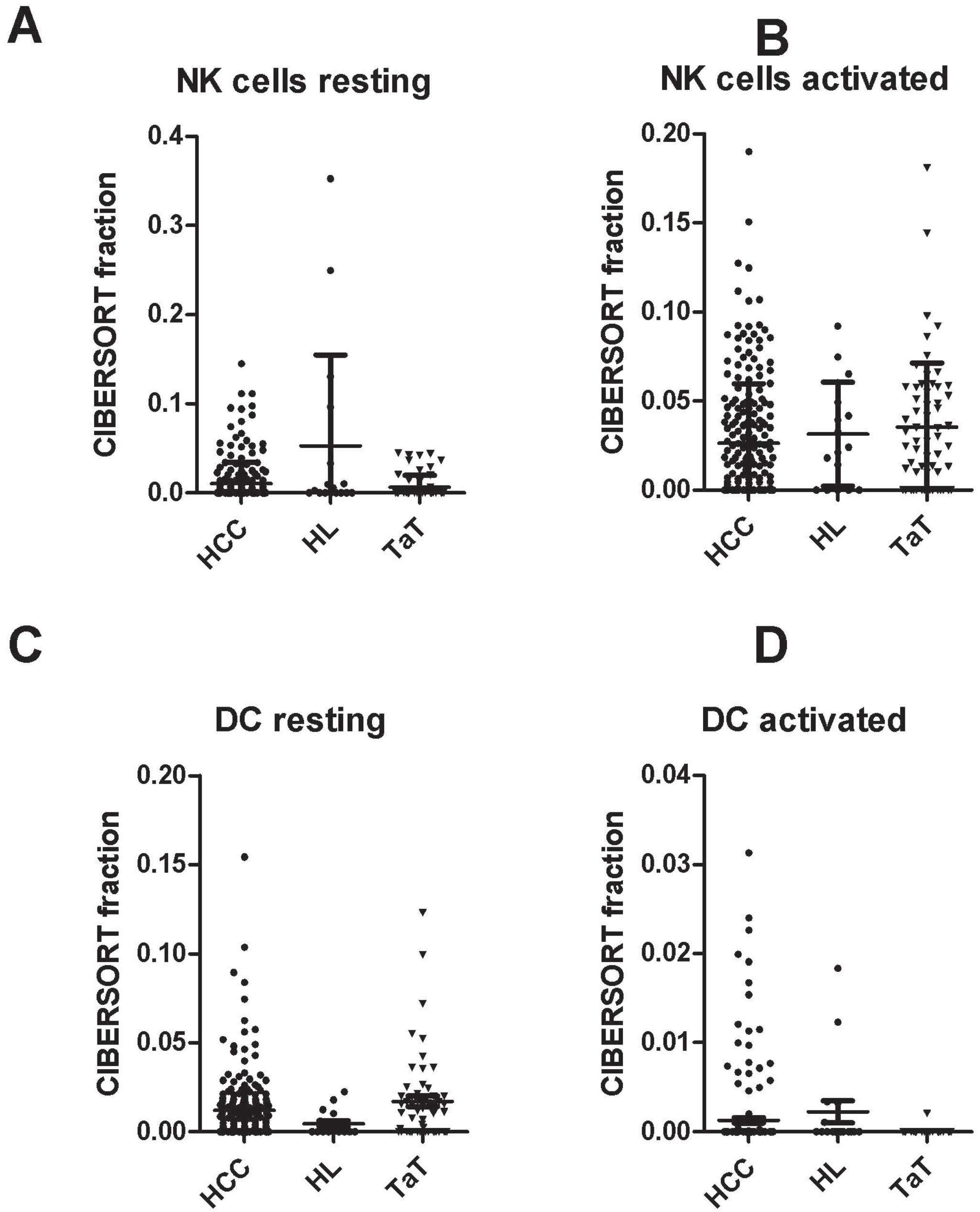

Supplementary Fig.1 . Natural Killer (NK) cells and dendritic cells (DC) in human HCC tumor tissue (HCC), adjacent tissue (TaT) and healthy liver (HL). CIBERSORT immune cell fractions were determined for each patient; each dot represents one patient. Mean values and standard deviations for each cell subset including resting NK cells (A), activated NK cells (B), resting dendritic cells (C) and activated dendritic cells (D) were calculated for each patient group and compared using one-way ANOVA. * $p<0.05$; ** $p<0.01$

Supplementary Fig. 2

	Healthy liver (n=17)		HCC tumor tissue (n=258)		Tumor adjacent tissue (n=46)	
	MC rest.	MC activ.	MC rest.	MC activ.	MC rest.	MC activ.
B_cells_naive	ns	ns	-0,11	ns	-0,275	0,352
B_cells_memory	ns	ns	-0,13	0,13	ns	ns
Plasma_cells	ns	-0,51	-0,30	ns	-0,322	ns
T_cells_CD8	ns	ns	ns	-0,15	-0,285	ns
T_cells_CD4_naive	ns	ns	ns	ns	ns	ns
T_cells_CD4_memory_resting	ns	ns	0,14	ns	ns	-0,384
T_cells_CD4_memory_activated	ns	ns	-0,26	ns	ns	ns
T_cells_follicular_helper	ns	-0,47	-0,41	0,15	-0,553	ns
Tregs	ns	-0,50	ns	ns	-0,257	ns
T_cells_gamma_delta	ns	ns	-0,19	0,16	ns	0,323
NK_cells_resting	0,56	-0,48	ns	ns	ns	ns
NK_cells_activated	ns	ns	0,21	ns	ns	ns
Monocytes	ns	0,50	0,28	-0,19	0,343	ns
Macrophages_M0	ns	0,52	0,42	-0,15	0,329	-0,263
Macrophages_M1	ns	ns	0,22	-0,164	ns	0,431
Macrophages_M2	ns	-0,51	-0,288	0,17	ns	ns
Dendritic_cells_resting	ns	ns		-0,159	-0,29	ns
Dendritic_cells_activated	ns	0,59	-0,232	0,197	ns	0,386
Eosinophils	ns	0,60	-0,347	0,291	-0,311	0,438
Neutrophils	ns	ns	-0,21	0,167	ns	ns

**bold**	p<0.05
**bold and underscored**	p<0.01
ns	no significant correlation
**red**	positive correlation ($r^2>0$)
**blue**	negative correlation ($r^2<0$)

suppl. Fig.2. Pearson correlation coefficients between resting /activated mast cells and other immune cell populations in healthy livers, HCC tumor tissues and tumor adjacent tissues. Cell populations for each patient were calculated by CIBERSORT as decribed in Materials and Methods.
